# Genetic Variation in Drug Targets: Are We Ready for the Era of Precision Medicinal Chemistry?

**DOI:** 10.1021/acsmedchemlett.5c00153

**Published:** 2025-04-11

**Authors:** Clinton G. L. Veale, Adrienne L. Edkins

**Affiliations:** † Department of Chemistry, 37716 University of Cape Town, Rondebosch, Cape Town 7701, South Africa; ‡ Biomedical Biotechnology Research Unit (BioBRU), Department of Biochemistry, Microbiology and Bioinformatics, 59100 Rhodes University, Makhanda 6139, South Africa

**Keywords:** Genetic Variation, Target Variation, Precision Medicine, Genetic-Guided Drug Discovery, Medicinal Chemistry

## Abstract

Natural genetic variations profoundly impact drug target interactions causing variations in in vitro biological data. The overall occurrence or “rare genetic variation” is common and enriched within population groups. Incorporating population-level genetic information earlier into the drug discovery pipeline would allow medicinal chemists to contribute to the precision medicine movement, designing drugs with more population relevance.

## Graphical abstract

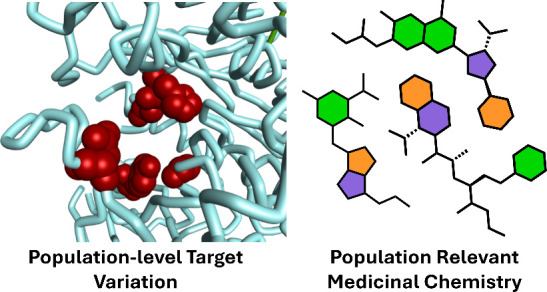

## The Genetic Revolution and Precision Medicine

The postgenomic era, and the associated advances in whole genome and proteome analyses, gave biologists their long-sought-after, broad-spectrum insight into the genetic causality of disease. This, in turn, ushered in a new paradigm for identifying targets potentially vulnerable to drugging. Disappointingly, these advancements did not immediately reinvigorate the drug discovery pipeline with the expected plethora of novel therapeutic options for unmet medical needs. This phenomenon was partly a result of the homogeneity of the reference genome, which did not account for population-level genetic variation.^1^

This recognition, enhanced by expanding genetic data repositories and increasingly sophisticated genomic and transcriptomic analysis methods, has precipitated growth in genome-wide association studies (GWAS). The integration of GWAS with multiomics approaches is providing population-level insight into genetic variations and their association to disease phenotype, including single nucleotide polymorphism (SNPs) in drug-associated genes, involved in both pharmacokinetic and pharmacodynamic processes.^2^ This pharmacogenomic approach has been fruitful in providing valuable insight into the causal link between genetic variation and population-level differences in drug disposition, levels of target expression, and links to efficacy and toxicity of drug therapies, thus ushering in the green shoots of the precision medicine era.^3^

In the context of drug development, this pharmacogenomic data is most commonly applied to retroactively optimize the dosing schedule of approved drug therapies. By its very nature, this largely excludes designers of novel medicines, including the medicinal chemistry community, from making meaningful contributions to the development of precision medicines.

In this Viewpoint, we highlight how natural genetic variations can profoundly impact drug–target interactions and argue that the impact of these variations on in vitro biological data would likely influence the trajectory of a novel medicinal chemistry campaign. We briefly discuss recent literature that suggests that, while individual variations are rare, their overall occurrence is common and often enriched within population groups. We therefore advocate for population-level genetic information to be incorporated earlier in the drug discovery pipeline,^4,5^ and, in so doing, inform the design of therapies to fit the genetic profile of a population subgrouping. Alternatively, by acknowledging this variation, small molecules are optimized with activity across common variants. This concept of genetically guided drug discovery at the level of the drug target would not only allow for the medicinal chemistry community to directly contribute to the development of precision medicines but also will be particularly powerful when developing medicines for diseases that disproportionately impact specific population groups.

## The In Vitro Impact of Target Variation

In addition to impacting drug disposition and target expression, genetic variations of target exons can impart subtle structural and conformational modifications to proteins, impacting catalytic sites, protein–protein interaction interfaces and small molecule binding sites.^6^ A handful of studies have identified exon variation in targets for numerous FDA-approved drugs attributing it as a factor which underpins variable clinical performance.^7,8^ However, outside of the realms of cancer chemotherapeutics^9^ and antimicrobials,^10^ which address pathological sequence alterations, the consequences of natural target genetic variation, and its impact as a variable for in vitro performance is largely underappreciated.

To demonstrate this phenomenon, Lauschke and co-workers recreated a series of in vitro bioassays, typical of those used in medicinal chemistry. Here, they assessed the variation in response of several FDA-approved drugs from three different therapeutic areas against the “wild-type” reference and naturally occurring genetic variants of their validated targets, namely Angiotensin Converting Enzyme, (ACE), tubulin β1 (TUBB1) and butylcholineesterase (BChE).^11^

With respect to the five ACE inhibitors, large fluctuations in biological response were observed for all the drugs against each natural target variant (Figure 1). Furthermore, these fluctuations were variant specific, following no discernible pattern of gain or loss of activity. The most notable fluctuation was observed for fosinopril (**1**), which at 10 μM displayed close to complete inhibition of the H520N ACE variant but was practically inactive against the Y530C ACE variant. Furthermore, the encouraging activity displayed for fosinopril against the H520N ACE variant was not mirrored by quinapril (**2**) which was functionally inactive against this same variant.

**Figure 1:**
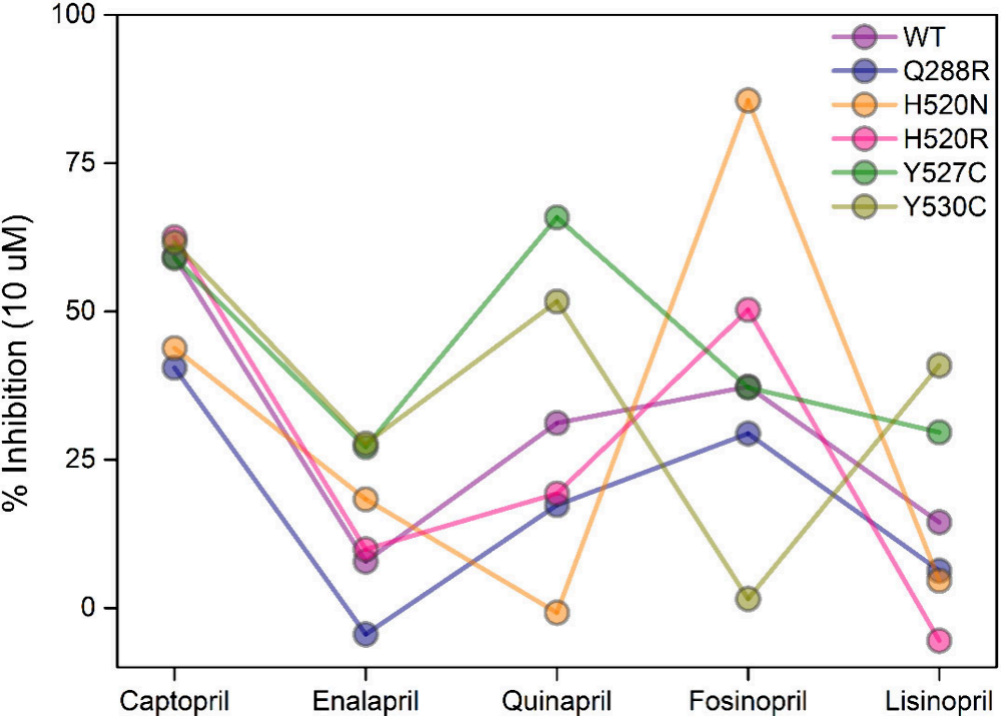
Variation in the response of five FDA-approved ACE inhibitors, against the reference and natural target variants. These data were derived from raw data supplied on request and presented in their current form with permission from the study PI.^11^

A similar effect was observed for the TUBB1 gene which encodes tubulin β1. The viability of cells expressing the reference “wild-type” tubulin was significantly reduced in the presence of 1 nM of the microtubule-destabilizing agent eribulin (**3**). While one natural tubulin β1 variant (C12Y) had limited impact on eribulin activity, six other natural tubulin β1 variants resulted in an approximately 4–8-fold reduction in activity at the same eribulin concentration (Figure 3). In the case of BChE, Lauschke and co-workers showed that the D98G variant conferred significant resistance to both tacrine (**4**) and rivastigmine (**5**).
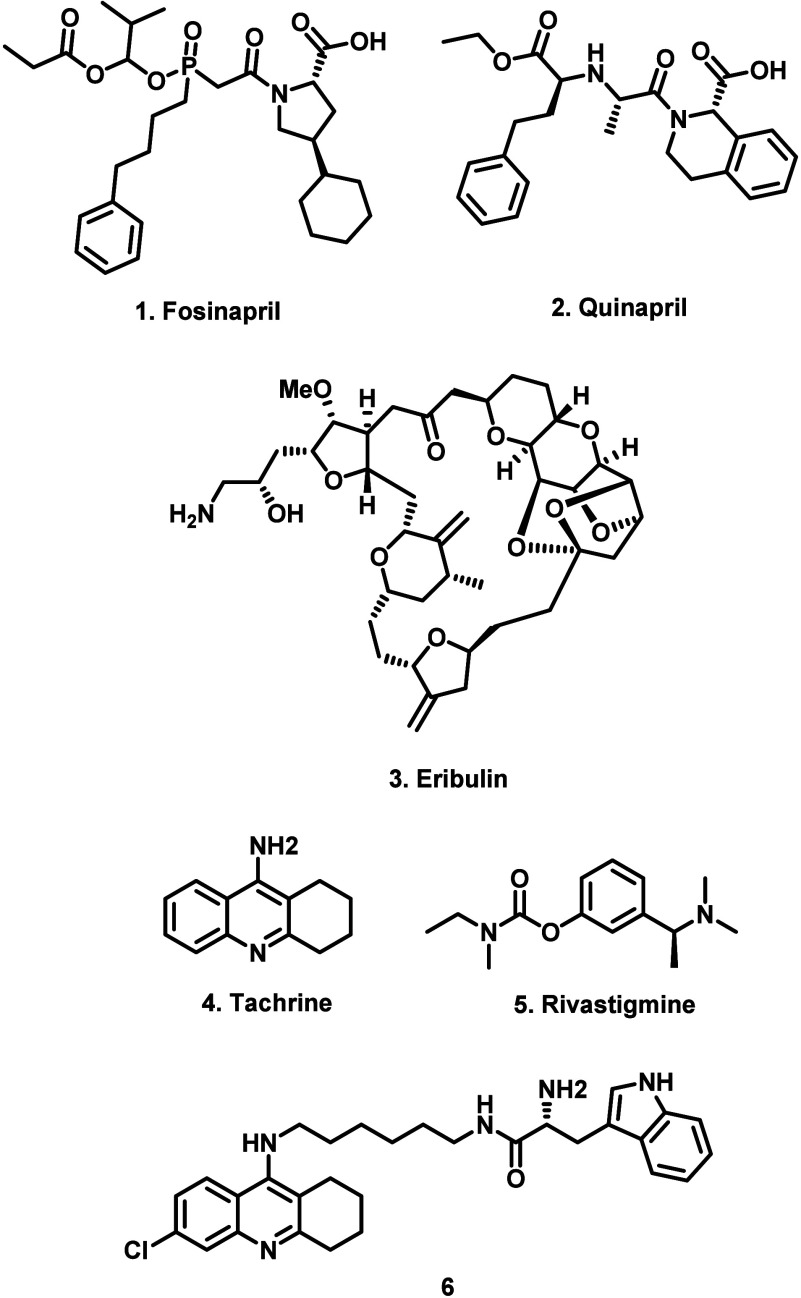


However, in an important demonstration of genetically guided drug development, Lauschke and co-workers screened a cohort of rationally designed flexible tachrine analogues identifying a handful of compounds (e.g., **6**) that recovered activity against both the reference and D98G variant of BChE. In a related study, Babu and co-workers showed through a real-time bioluminescence resonance energy transfer (BRET) assay, that three previously unreported naturally occurring polymorphs of the μ-opioid receptor altered receptor signaling, and receptor responses to a variety of FDA-approved full agonists, partial agonists and antagonists.^12^ A structural overlay of the positions of all the examples described in these studies showed that, apart from the R241W variation of tubulin β1, all of these pharmacologically relevant variations occurred in very close proximity to the small molecule binding site (Figure 2), and were offered as a compelling structural rationalization for the observed variation in in vitro bioassay performance.

**Figure 2:**
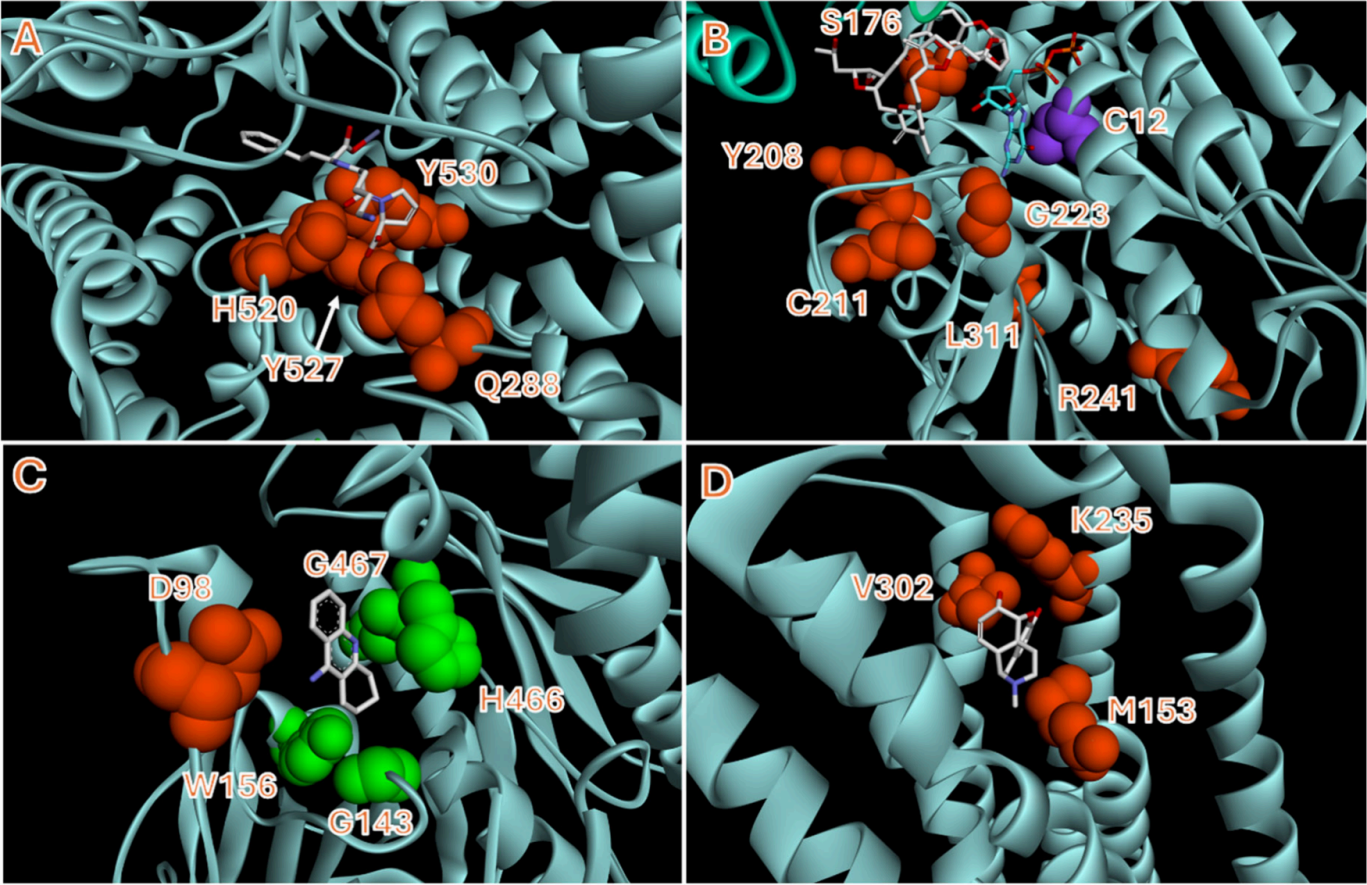
Structural overlay of FDA-approved inhibitors bound to their targets. Residues that have been identified as positions of natural genetic variation are highlighted. These are all found near the drug binding cleft and, in most instances, impacted the in vitro performance of their accompanying drugs. Numbering of residues is linked to those used in refs 11 and 12 and may not correlate with those provided in the PDB structure. (A) Lisinopril bound to ACE (PDB ID 1O86), (B) Eribulin bound to tubulin β1 (PDB ID 5JH7). The C12 residue highlighted in purple had no impact on eribulin activity. (C) Tachrine bound to BChE (PDB ID 4BDS). The D98 residue conveyed significant resistance to tacrine and rivastigmine. The residues highlighted in green reduced BChE activity making bioassay unreliable. (D) Morphine bound to the μ-opioid receptor (PDB ID 8EF6).

## How Abundant Are These Variations and What Are the Implications?

Several studies have shown that, while individual variants are rare, their overall occurrence is abundant within human populations, estimated to occur, on average, in 1 in 17 bases and tending to be more prevalent in functional genes.^13^ Genetic variation in drug-related genes have been predicted to be present in approximately four out of five individuals,^8^ with one in six individuals carrying at least one variant in the binding pocket of an FDA-approved drug. It is important to note that this information is derived from examples for which protein–ligand binding information is available and only considered those SNPs at regions in close proximity to drug binding interfaces and not any distal mutations that may allosterically alter protein conformation. Taken together, this suggests that the frequency of binding site variance is likely more widespread.^11^

Importantly, this variability shows evidence of ethnogeographic localization (Figure 3) with an approximately 3-fold enrichment of binding site variation observed within discrete population groups.^8,11,13^ One analysis that assessed the impact of human polymorphisms on drug–protein interactions found that, within ethnogeographically distinct population groups, individuals carried, on average, ∼1 SNP, which could probably or possibly affect the drug–target interaction of FDA-approved drugs.^14^ Their data further suggested that this number increased to ∼8 SNPs per individual when exploring FDA experimental drugs.^14^ This pattern indicates that FDA approvals favor molecules whose target interactions are less susceptible to genetic variation. Given the paradigms of the current drug discovery, this outcome is reasonable. However, this pattern also suggests that, without considering target variation, efforts to clinically exploit new areas of chemical space will continue to be hampered.

**Figure 3:**
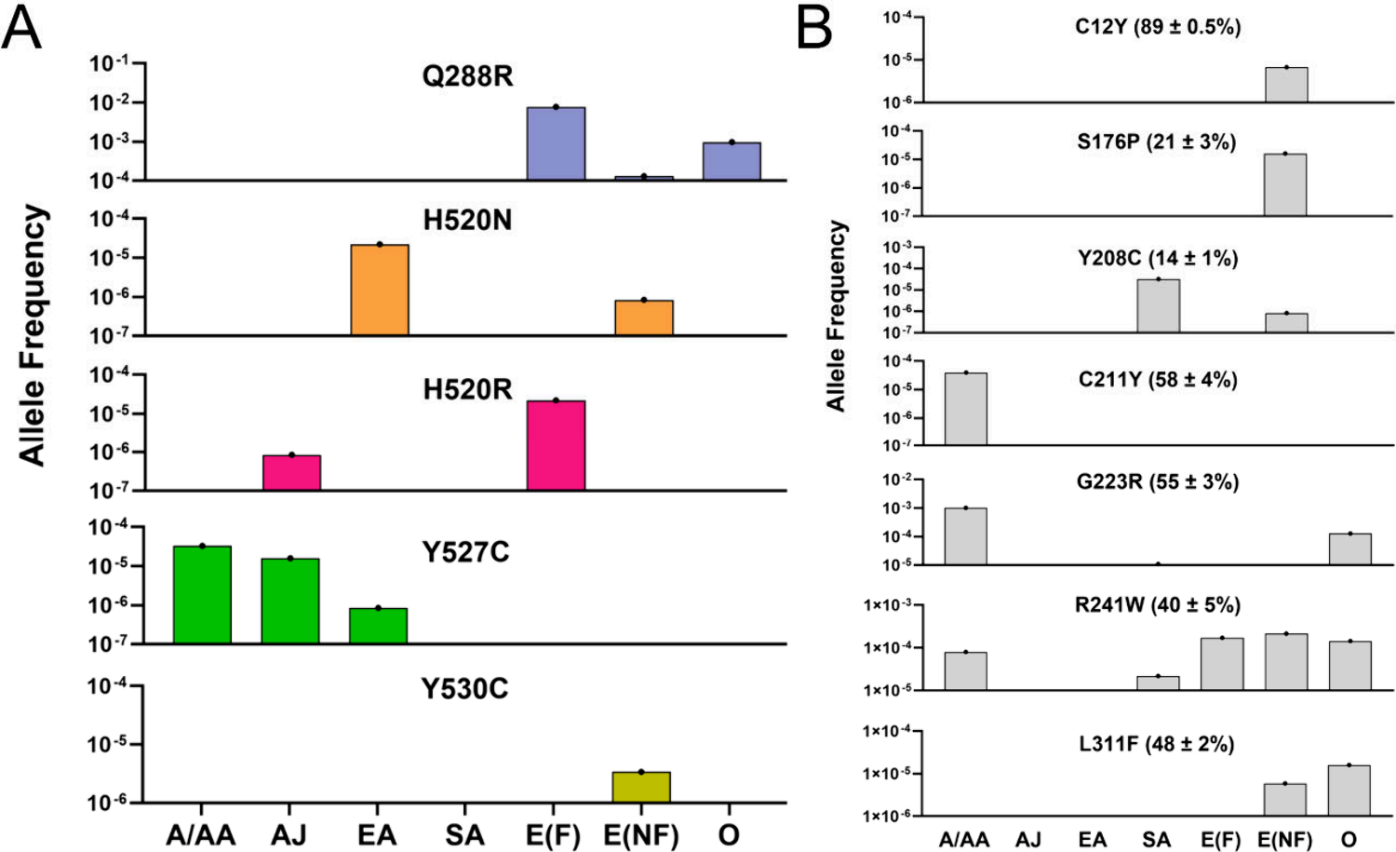
Allele frequency for variants with altered drug responses in different population groups for (A) ACE and (B) TUBB1 variants. For panel (A), the alleles shown correspond to the responses to ACE inhibitors shown in Figure 1. For panel (B), the values in brackets represent the percentage cell viability for cells expressing tubulin β1 with the given variation in response to treatment with 1 nM eribulin. In comparison, eribulin reduced viability by 92% ± 0.6% in cells with the reference tubulin β1 sequence.^11^ Allele frequencies were retrieved from gnomAD. [Legend: A/AA, African/African American; AJ, Ashkenazi Jew; EA, East Asian; SA, South Asian; E­(F), European Finnish; E­(NF), European Non-Finnish; O, other populations.]

An additional consideration is the lack of global diversity within genetic databanks.^15^ This Eurocentricity in the available data makes it likely that the extent of target variation, and its pharmacological implications, particularly within underrepresented ethnic groups, is underestimated.

For example, one African genomic survey identified over 3 million undescribed genetic variants. What made this all the more extraordinary, was that this study was only conducted on 426 individuals from 15 African countries.^16^ This African genetic diversity is reflected in genes involved in both pharmacokinetic and pharmacodynamic processes.^17^ The high proportion of suboptimal therapeutic outcomes and adverse drug reactions experienced by African patients is commonly attributed to pharmacokinetic gene variations.^18^ However, the underappreciated impact of target variation cannot be excluded as a contributing factor and may be a key piece of the puzzle for the medicinal chemistry community when addressing therapies for neglected diseases.

## Conclusions and Recommendations

The application of the concept of pharmacogenomics has primarily focused on the late stages of drug development and optimization of drug therapies postapproval. However, the handful of studies that have investigated the impact of drug target variation on small molecule efficacy in vitro inadvertently opened the doorway for drug designers working at the earliest stages of the pipeline to contribute. In light of these studies, it is worth considering how a medicinal chemistry campaign may have deviated had it incorporated a natural target variant, rather than the reference “wild type”. Would the amino acid change, which subtly varies the precise requirements for optimal target interaction impact the selection of a screening hit or alter the trajectory of a small molecule optimization campaign? Could differences in biological activity ultimately influence compound prioritization, and preclinical candidate selection? In addition to direct binding site interactions, it is likely that this same effect would be observed in distal variations that allosterically alter the binding site, as well as at protein–protein interaction interfaces and other classes of challenging drug targets, to which recent technological advances are providing us access. The ethnogeographic enrichment of these variations, and the biases in the available genetic data, not only suggest that the impact of target variation is underestimated but also provides a unique opportunity for genetically guided drug discovery to make inroads into the abundance of neglected diseases that disproportionately impact the Global South.^5,19^ This phenomenon is also not limited to noncommunicable disease. Normal host genetic heterogeneity has a significant influence on host–pathogen interactions, impacting infectious disease susceptibility and virulence. The unique selection environments created by host–pathogen interaction also promotes the emergence of genetically diverse microbial strains, independently of antimicrobial induced drug exposure.^20^

Given the increasing recognition of the role of ethnogeographic genetic diversity in health, coupled with the precision medicine movement, has the time arrived for population-level genetic diversity to be front-loaded into the medicinal chemistry phase of drug discovery? While this Viewpoint has focused on examples using recombinant proteins, it would include the generation of more-relevant human cell lines,^21^ and the use of clinically relevant microbial strains, while being particularly amendable to in silico workflows, particularly given the advances in structural prediction tools like AlphaFold. Without being naïve about the likelihood of adverse effects, and idiosyncratic responses, could the medicinal chemistry community play a leading role in developing medicines with more relevance, and efficacy for groups that the medicines were initially intended?

## Supplementary Material



## Notes

Lay summary (PDF)
