# Supplementary material for: Genetic Variation in Drug Targets: Are We Ready for the Era of Precision Medicinal Chemistry?
Source: ACS Med Chem Lett. 2025 Apr 11;16(5):706–10. doi: 10.1021/acsmedchemlett.5c00153 (PMC12067103; doi:10.1021/acsmedchemlett.5c00153)
Supplement: Supplementary file 1 [file ml5c00153_si_001.pdf]

Lay summary

Genetic differences between individuals can affect how drugs interact with their targets, leading to variations in their efficacy. Although some genetic variations are considered “rare”, they are actually quite common within specific populations. By considering genetic diversity earlier in the drug development process, medicinal chemists can design drugs that are more effective and more relevant for different population groups thus, helping to advance precision medicine.
